# Syndrome coronaire aigu avec sus-décalage du segment ST chez un jeune à coronaires saines révélant une myocardite aigue

**DOI:** 10.11604/pamj.2018.29.80.11153

**Published:** 2018-01-26

**Authors:** Houssam Laachach, Bachrif Mohamed, Ilham Benahmed, Alaa Fliti, Noha El Ouafi

**Affiliations:** 1 Service de Cardiologie, CHU Mohammed 6, Université Mohammed Premier, Oujda, Maroc

**Keywords:** Syndrome coronarien aigu, jeune, myocardite aigue, IRM, Acute coronary syndrome, young, acute myocarditis, MRI

## Abstract

Les syndromes coronariens aigus peuvent parfaitement survenir chez l'adulte jeune, les étiologies non-athéromateuses peuvent en être le mécanisme. Nous rapportons le cas d'un jeune homme admis dans un tableau de coronaropathie aigue avec sus-décalage du segment ST systématisé chez qui la coronarographie était sans anomalies et l'imagerie par résonnance magnétique a confirmé une myocardite aigue ayant bien évoluée sous traitement médical.

## Abstract (en)

Acute coronary syndromes may occur in young adults and have a non-atheromatous origin. We report the case of a young man admitted with acute coronary artery disease with systematic ST-segment elevation. Coronarography showed no abnormality and MRI confirmed acute myocarditis. Outcome was favorable under medical treatment.

## Introduction

Chez le sujet jeune sans facteurs de risques cardiovasculaire, l'athérosclérose peut ne pas être le mécanisme fondamental responsable de la survenue d'un syndrome coronarien aigu. La myocardite est une des étiologies des coronaropathies aigues à coronaires saines qui prédominent préférentiellement chez le sujet jeune nécessitant une imagerie clinique adaptée et pouvant engendrer le pronostic vital.

## Patient et observation

**Figure 1:**
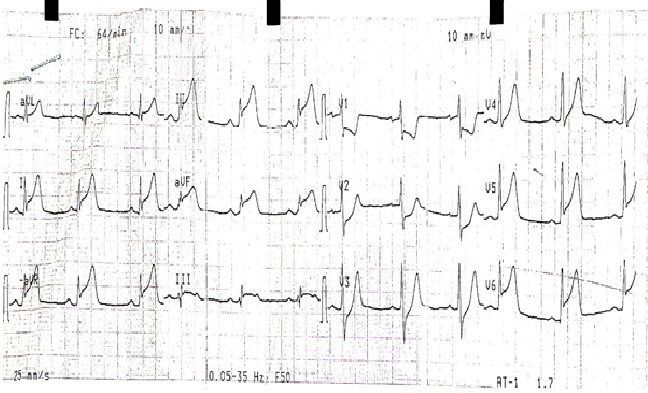
Tracé ECG avec sus-décalage ST latéral et inferieur

**Figure 2:**
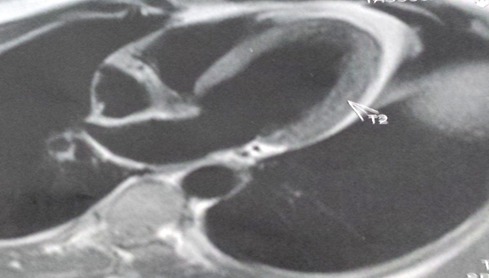
Image IRM d'hyperhémie myocardique significatives d'une myocardite aigue

**Figure 3:**
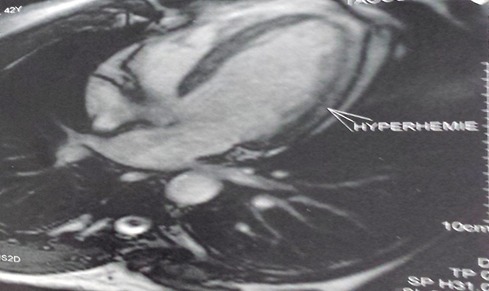
Image IRM (après injection de Gadolinium) d'hyperhémie myocardique significatives d'une myocardite aigue

Un jeune patient de 39 ans sans facteurs de risque cardio-vasculaires ni antécédents pathologiques, était admis à H2 du début d'une douleur thoracique rappelant un syndrome coronaire aigu. L'état hémodynamique était stable, l'examen clinique était sans anomalies et le tracé électrocardiographique s'est inscrit un sus-décalage ST persistant en latéral et en inferieur avec image en miroir (Figure 1). Le bilan biologique a objectivé un syndrome inflammatoire avec élévation des enzymes de nécrose myocardique, sans perturbation du bilan lipidique. Le patient a été thrombolysé sans autant avoir faire l'apparition des signes électro-cliniques de succès. L'echo-cœur trans-thoracique avait objectivé des hypokinésies avec une dysfonction modérée de la fonction systolique du ventricule gauche et la coronarographie était sans anomalies. L'IRM cardiaque a confirmé une myocardite (Figure 2,Figure 3) et le bilan de thrombophilie et des sérologies virales communes revenait normal. L'évolution sous anti-inflammatoire avec un inhibiteur de l'enzyme de conversion a été marqué par la régression des symptômes cliniques et la récupération ad integrum de la fonction systolique du ventricule gauche.

## Discussion

Lors des douleurs thoraciques angineuses associées à des modifications significatives du tracé électrocardiographique ou à une augmentation des enzymes de nécrose myocardiques, le diagnostic de l'ischémie aigue du myocarde se pose décidément sauf dans de rares cas où son élimination se base sur la coronarographie immédiate [1,2]. La myocardite est une pathologie insidieuse, son incidence reste mal précisée [3]. Elle est considérée pauci-symptomatique, pouvant évoluer vers une cardiomyopathie chronique ou causer une mort subite avant l'âge de 40 ans [1,3]. Ses manifestations cliniques peuvent être très discrètes ou mimer un syndrome coronarien aigu [4]. Les troubles de repolarisation électrocardiographiques type sus-décalage du segment ST peuvent être diffus, avec élévation des enzymes myocardiques notamment la créatine kinase et les troponine I et C sans autant en être synonyme de gravité [5]. L'échocardiographie en mode 2D [6] et plus perfectionnement le mode strain longitudinal [7] apporte une aide indéniable au diagnostic positif, elle étudie la fonction et la cinétique segmentaire des parois ventriculaires. La coronarographie élimine les coronaropathies aigues comme au cours d'exploration de notre cas. Une biopsie myocardique est de réalisation extrêmement rare en pratique courante, mais reconnue comme l'examen de certitude apportant le diagnostic anatomo-pathologique d'une myocardite. L'IRM cardiaque en différentes séquences est actuellement le gold standard pour le diagnostic positif [8]. L'évolution bénigne comme l'est remarquée dans notre cas n'est pas une certitude, compte tenu de la probabilité de progression vers une cardiomyopathie dilatée. Une telle progression est énoncée par l'apparition d'une dilatation précoce du ventricule gauche et la non récupération de sa fonction systolique. Bien que les causes en soient multiples, l'étiologie infectieuse, notamment virale, en constitue la principale étiologie. Si le virus Coxsackie B et l'ensemble des entérovirus sont couramment impliqués dans les pays développés et le VIH en Afrique sub-saharienne, plusieurs études récentes font état du rôle méconnu du virus HHV6 et du parvovirus B19 voire de l'association des deux [9]. Le traitement se base en premier lieu sur les soins de soutien d'une éventuelle instabilité hémodynamique, sur les différentes mesures thérapeutiques des signes d'insuffisance cardiaque avec prévention du remodelage ventriculaire [3]. L'utilisation de la digoxine à très faible dose nécessite une grande précaution et peut être nuisible [10]. Le traitement anti-inflammatoire améliore les signes inflammatoire et aide à la guérison ; l'administration des immunosuppresseurs ainsi que l'immunoglobuline n'est pas de routine [11].

## Conclusion

La suspicion clinique d'une myocardite aigue, particulièrement dans un contexte de syndrome coronarien aigu, implique une exploration radiologique belle et bien via une IRM myocardique et une prise en charge rapide et armée évitant la mise en jeu précoce du pronostic vital et la progression vers une cardiomyopathie dilatée.

## Conflits d’intérêts

Les auteurs ne déclarent aucun conflit d'intérêts.

## References

[1] Mahajan N, Mehta Y, Rose M, Shani J, Lichstein E (2006). Elevated troponin level is not synonymous with myocardial infarction. Int J Cardiol..

[2] Nageh T, Sherwood RA, Wainwright RJ, Shah AM, Thomas MR (2005). The clinical relevance of raised cardiac troponin I in the absence of significant angiographic coronary artery disease. Int J Cardiol..

[3] Feldman M, McNamara D "Myocarditis," .The New England Journal of Medicine. 2000;.

[4] Dec GW Jr, Waldman H, Southern J, Fallon JT, Hutter AM Jr, Pala-cios I (1992). Viral myocarditis mimicking acute myocardial infarction. J Am Coll Cardiol..

[5] Gilotra NA, Minkove N, Bennett MK, Tedford RJ, Steenbergen C, Judge DP, Halushka MK, Russell SDJ (2016). Lack of Relationship Between Serum Cardiac Troponin I Level and Giant Cell Myocarditis Diagnosis and Outcomes. Card Fail..

[6] Goitein O, Matetzky S, Beinart R, Di Segni E, Hod H, Bentancur A, Konen E (2009). Acute myocarditis: noninvasive evaluation with cardiac MRI and transthoracic echocardiography. AJR Am J Roentgenol..

[7] Hsiao JF, Koshino Y, Bonnichsen CR, Yu Y, Miller FA Jr, Pellikka PA, Cooper LT Jr, Villarraga HR (2013). Speckle tracking echocardiography in acute myocarditis. Int J Cardiovasc Imaging..

[8] Nucifora G, Gillebert C, Selvanayagam JB (2016). Value of novel cardiac magnetic resonance indices for the diagnosis of acute myocarditis: Left ventricular mechanics and parametric mapping imaging. Int J Cardiol..

[9] Mahrholdt H, Wagner A, Deluigi CC (2006). Presentation, patterns of myocardial damage and clinical course of viral myocarditis. Circulation..

[10] Matsumori A, Igata H, Ono K (1999). High doses of digitalis increase the myocardial production of proinflammatory cytokines and worsen myocardial injury in viral myocarditis: a possible mechanism of digitalis toxicity. Jpn Circ J..

[11] Heart Failure Society of America (HFSA) practice guidelines (1999). HFSA guidelines for management of patients with heart failure caused by left ventricular systolic dysfunction: Pharmacologic approaches. J Card Fail..

